# The Role of Granulocyte Colony-Stimulating Factor in Endometrial Preparation for Embryo Implantation in In Vitro Fertilization

**DOI:** 10.3390/life16020351

**Published:** 2026-02-18

**Authors:** Charalampos Voros, Fotios Chatzinikolaou, Georgios Papadimas, Iwakeim Sapantzoglou, Aristotelis-Marios Koulakmanidis, Vaitsis Dimitrios, Diamantis Athanasiou, Vasiliki Kanaka, Kyriakos Bananis, Antonia Athanasiou, Aikaterini Athanasiou, Ioannis Papapanagiotou, Charalampos Tsimpoukelis, Athanasios Karpouzos, Maria Anastasia Daskalaki, Nikolaos Kanakas, Marianna Theodora, Nikolaos Thomakos, Panagiotis Antsaklis, Dimitrios Loutradis, Georgios Daskalakis

**Affiliations:** 1 The 1st Department of Obstetrics and Gynecology, ‘Alexandra’ General Hospital, National and Kapodistrian University of Athens, 80 Vasilissis Sofias Avenue, 11528 Athens, Greece; kimsap1990@hotmail.com (I.S.); aristoteliskoulak@gmail.com (A.-M.K.); vaitsisdim@gmail.com (V.D.); vasilikikanaka@outlook.com (V.K.); tsimpoukelischa@gmail.com (C.T.); thanoskarpouzosdr@hotmail.com (A.K.); md181341@students.euc.ac.cy (M.A.D.); martheodr@gmail.com (M.T.); thomakir@hotmail.com (N.T.); panosant@gmail.com (P.A.); gdaskalakis@yahoo.com (G.D.); 2 Laboratory of Forensic Medicine and Toxicology, School of Medicine, Aristotle University of Thessaloniki, 54124 Athens, Greece; fotischatzin@auth.gr (F.C.); loutradi@otenet.gr (D.L.); 3 Athens Medical School, National and Kapodistrian University of Athens, 15772 Athens, Greece; dr.georgepapadimas@gmail.com (G.P.); gpapamd@hotmail.com (I.P.); 4 King’s College Hospital NHS Foundation Trust, London SE5 9RS, UK; diamathan16@gmail.com (D.A.); kyriakos.bananis@nhs.net (K.B.); 5 IVF Athens Reproduction Center, 15123 Maroussi, Greece; antoathan16@gmail.com (A.A.); diamathan17@gmail.com (A.A.); 6 School of Medicine, European University Cyprus, Nicosia 2404, Cyprus; nkanakas@icloud.com; 7 Fertility Institute-Assisted Reproduction Unit, Paster 15, 11528 Athens, Greece

**Keywords:** granulocyte colony-stimulating factor, endometrial receptivity, thin endometrium, recurrent implantation failure, in vitro fertilization, immune stromal interaction, embryo implantation

## Abstract

Granulocyte colony-stimulating factor (G-CSF) has been suggested as a supplementary approach for endometrial preparation in IVF. Clinical results continue to be inconsistent. This narrative review synthesises molecular and clinical information to elucidate the function of G-CSF in modifying endometrial receptivity and to identify patient categories most likely to benefit. A thorough assessment was conducted on published research on G-CSF administration in women with treatment-resistant thin endometrium, recurrent implantation failure, and unselected IVF populations. The research demonstrates that G-CSF has phenotype-dependent effects. Improvements in pregnancy and live birth rates are inconsistent and seem dependent on the reversibility of underlying tissue disease; nevertheless, G-CSF reliably increases endometrial thickness in instances of thin endometrium and may restore eligibility for transfer. G-CSF improves implantation and early pregnancy outcomes in repeated implantation failure patients without modifying endometrial morphology, indicating a functional mechanism linked to immune-stromal synchronisation rather than structural expansion. In contrast, randomised controlled studies show no therapeutic benefit in unselected IVF groups. Discrepancies in research outcomes may mostly be attributed to variations in patient phenotype, initial endometrial function, and the therapy setting. Thus, G-CSF should be considered a specific approach for endometrial conditioning rather than just a supplementary component of IVF.

## 1. Introduction

Notwithstanding significant advancements in ovarian stimulation methods, embryo culture environments, and embryo selection technologies, implantation failure persists as the foremost unsolved issue in in vitro fertilization [1]. Implantation often fails, even with morphologically or genetically viable embryos, highlighting the critical significance of endometrial receptivity. During this brief period of endometrial receptivity, it undergoes synchronised molecular, cellular, and immunovascular reprogramming that facilitates the embryo’s attachment, invasion, and adherence. This technique is very sensitive to variations and must be executed simultaneously, rendering traditional endocrine preparation ineffective for all individuals [2].

The administration of oestradiol and progesterone is the principal clinical approach for endometrial preparation, with endometrial thickness acting as a practical indicator of readiness [3]. However, growing clinical and molecular data indicates that thickness is not a reliable singular indication of receptivity. Certain women with seemingly adequate endometrial thickness have recurrent implantation failure despite several transfers of high-quality embryos, whereas women with treatment-resistant thin endometrium often demonstrate an unresponsive reaction to supraphysiologic oestrogen exposure [4]. These phenotypes highlight the need for therapies that prioritise functional endometrial conditioning above mere morphology, since implantation failure often signifies qualitative defects in endometrial programming rather than inadequate proliferation [5].

Endometrial receptivity transpires at the molecular level via the amalgamation of progesterone-dependent transcriptional mechanisms with immunological tolerance, microvascular adaptability, stromal differentiation, and epithelial remodelling. The decidualisation of endometrial stromal cells is a crucial component of this process [6]. It is governed by meticulously regulated transcriptional networks, including progesterone receptor signalling, HOXA10, FOXO1, CEBPβ, and members of the STAT family. These substances facilitate the development of a decidual phenotype in stromal cells, enabling them to assist trophoblast invasion via the coordination of alterations in the cytoskeleton, metabolism, and chromatin. Despite sufficient progesterone levels, disruption of these mechanisms leads to reduced decidual competence and unsuccessful implantation [7].

The functional reprogramming of the luminal epithelium is shown by the loss of apical polarity, the reorganisation of tight and adherens junctions, and the regulated expression of adhesion molecules like integrins and cadherins that facilitate blastocyst attachment [8]. Stromal signalling is closely associated with this epithelial transition, which is further influenced by paracrine signals from the immunological and endothelial compartments. Thus, in clinically confusing situations, the disruption of epithelial stromal communication might dissociate morphological traits from functional receptivity, leading to implantation failure [9].

Endometrial immune adaptability is a significant component influencing receptivity. During the implantation window, the uterine immunological milieu transitions to a carefully regulated tolerogenic state. Macrophages polarise to an M2-like phenotype, promoting tissue remodelling and angiovascular stability; regulatory T cells multiply to enhance immunological tolerance [10]. Uterine natural killer cells undergo functional maturation that prioritises vascular remodelling over cytotoxicity. These immunological systems are closely linked to vascular adaptability and stromal decidualisation, rather than operating independently. This degree of dysregulation may result in excessive inflammation or insufficient immunological support, both detrimental to effective implantation [11].

In this complex biological environment, growth factor-based methods that restore endometrial functionality instead of promoting nonspecific proliferation have received heightened interest. Granulocyte colony-stimulating factor is a multifunctional cytokine primarily recognised for its function in haematopoiesis [12]. Nonetheless, it significantly impacts the immune system and the remodelling of peripheral tissues. The human endometrium exhibits cycle-dependent expression of both G-CSF and its receptor, suggesting a physiological function in implantation processes and offering a mechanistic basis for its therapeutic investigation in assisted reproduction [13].

The JAK–STAT pathway is the primary mechanism via which G-CSF transmits signals at the molecular level, with STAT3 serving as the principal downstream effector. STAT3 is crucial for endometrial biology since it integrates progesterone signalling with decidualisation, immunological tolerance, and vascular adaptability [14]. Upon activation, STAT3 directly connects G-CSF signalling with critical decidual transcriptional processes by promoting the transcription of implantation-related genes, such as HOXA10. The activation of STAT3 improves decidual stability throughout the peri-implantation period by increasing survival, differentiation, and resistance to apoptosis in endometrial stromal cells. These data indicate that G-CSF affects stromal functional competency and acts as a proliferative stimulant [14].

Besides its impact on the stroma, G-CSF significantly influences the immunological microenvironment of the endometrium. G-CSF facilitates the immune system’s acceptance of foreign substances and mitigates excessive inflammatory responses via cytokine-mediated signalling [15]. It facilitates controlled trophoblast invasion by augmenting regulatory T-cell activity and encouraging macrophage polarisation towards an anti-inflammatory, reparative phenotype. Despite the limited expression of traditional G-CSF receptors by uterine natural killer cells, indirect regulation via cytokine networks likely enhances immune-vascular interaction and vascular remodelling during implantation [16].

Vascular modulation is another significant mechanism via which G-CSF operates. G-CSF seems to enhance endothelial survival, maintain junctional integrity, and stabilise microvasculature rather than provoke indiscriminate angiogenesis [17]. Clinical investigations indicate that improvements in endometrial blood flow indices, vascularization flow index, and endometrial volume support the idea that the principal vascular impact of intrauterine G-CSF perfusion is functional optimisation rather than vessel growth. In thin or inadequately perfused endometrium, improved perfusion and endothelial stability may promote oxygen supply, stromal responsiveness, and decidual expansion [18].

This mechanistic specificity is apparent in clinical outcomes. The delivery of intrauterine G-CSF has consistently correlated with increased endometrial thickness and, in some cases, the preservation of otherwise failed cycles in women with treatment-resistant thin endometrium [13]. However, increases in thickness may not always result in higher live birth rates, highlighting that morphological improvement alone is inadequate. Certain investigations have shown a correlation between the administration of G-CSF both intrauterinely and extrauterinely and increased rates of implantation and clinical pregnancy in women experiencing recurrent implantation failure. This indicates that immunological and stromal reprogramming may be particularly significant for this cohort of women [19]. In contrast, randomised studies in unselected IVF populations have shown no benefit, suggesting that G-CSF does not improve receptivity beyond an already excellent physiological baseline.

Collectively, our data support a concept whereby G-CSF acts as a context-dependent endometrial conditioning agent, with its effectiveness contingent upon pre-existing endometrial dysfunction, timing in relation to the implantation window, and manner of delivery. The biological effects include three interrelated axes: stability of the endometrial microvasculature, modification of immunological tolerance pathways, and augmentation of decidual transcriptional programs. The variety and inconsistent outcomes reported in clinical investigations are largely due to the oversight of these aspects. Our narrative review aims to present the most recent clinical and translational findings on the role of granulocyte colony-stimulating factor in endometrial preparation for embryo implantation during in vitro fertilisation. We want to clarify the mechanistic foundations of G-CSF activity, identify patient groups most likely to benefit, and outline the limitations that now impede its regular clinical use by integrating molecular signalling pathways with clinical trial data. Transitioning from experiential endometrial conditioning strategies in assisted reproduction to logical, tailored approaches necessitates the significance of this mechanistically grounded strategy.

## 2. Materials and Methods

This narrative review was conducted to gather clinical and translational information about the function of granulocyte colony-stimulating factor in endometrial preparation for embryo implantation during in vitro fertilisation, via a systematic and repeatable literature search. To guarantee thoroughness, a complete search of the PubMed/MEDLINE database was followed by a focused evaluation of reference lists from pertinent publications and systematic reviews. The search strategy encompassed terminology pertinent to granulocyte colony-stimulating factor and endometrial receptivity, including “granulocyte colony-stimulating factor,” “G-CSF,” “endometrium,” “endometrial receptivity,” “thin endometrium,” “recurrent implantation failure,” “embryo transfer,” and “in vitro fertilisation.” Boolean operators were used to enhance the precision and sensitivity of the search.

The search included papers published from the inception of the database to March 2025. Randomised controlled trials, observational studies, translational research, and mechanistic investigations pertinent to endometrial biology were included without initial restrictions on study design. Only English-language publications were taken into account. Conference abstracts without complete articles were discarded due to insufficient methodological depth. Studies that satisfied at least one of the following criteria were deemed eligible: (i) Research concentrating on women with treatment-resistant thin endometrium or recurrent implantation failure; (ii) translational or mechanistic investigations analysing G-CSF signalling pathways, receptor expression, or downstream molecular effects in endometrial tissue or endometrial stromal cells; or (iii) systematic reviews and meta-analyses offering synthesised evidence pertinent to the clinical utilisation of G-CSF in assisted reproduction.

Studies that investigated the effects of embryo culture without endometrial endpoints, concentrated only on haematologic indicators of G-CSF without relevance to reproductive tissues, or shown inadequate methodological clarity were rejected. Case reports and short case series were included just when they investigated carefully chosen clinical situations not represented in bigger studies or offered mechanistic insights. We examined the titles and abstracts identified in the first search to assess their relevance to implantation biology and endometrial preparation. Upon indication of probable eligibility in the abstracts, the entire texts were then examined. The selection of research was determined by their pertinence to the basic conceptual framework of this review, particularly the function of G-CSF as a modulator of endometrial functional competence rather than a nonspecific proliferative agent. Discrepancies in research relevance were addressed by a rigorous assessment of biological plausibility, clinical context, and contributions to mechanistic knowledge.

Studies were weighted based on their relevance to particular thematic themes, such as stromal decidualisation, immunological regulation, vascular adaptability, administration route and timing, and patient profile, rather than strict inclusion criteria. This method preserved conceptual consistency while enabling the integration of diverse evidence. We extracted data from each research, including details on the study design, patient demographics, indications for G-CSF administration, method and timing of delivery, main and secondary reproductive outcomes, and documented effects on endometrial parameters. Mechanistic data were collected where feasible, including details on vascular indices, immune cell modulation, transcriptional regulators, and signalling pathways. Particular emphasis was placed on the distinctions among fresh and frozen embryo transfer cycles, decreased endometrial thickness, recurrent implantation failure, and unselected IVF cohorts. A quantitative meta-analysis was not performed due to inconsistencies in research designs, outcomes, and administration techniques. The data were instead summarised narratively, focusing on identifying mechanistic patterns that might explain variances in clinical outcomes. Clinical data were examined in relation to basic endometrial biology to connect trial outcomes with molecular and cellular causes.

Our review was deliberately structured as a narrative synthesis to integrate clinical, translational, and mechanistic information that conventional meta-analytic approaches would inadequately address. Our work emphasises mechanistic interpretation and conceptual integration above effect size estimates; however, previous systematic reviews and meta-analyses were taken into account. Clear search strategies and precise definitions of research inclusion criteria reduced the limitations of narrative reviews, such as possible selection bias. This study mainly seeks to elucidate the biological basis, contextual efficacy, and outstanding questions about the use of G-CSF as an endometrial conditioning agent, rather than its conventional clinical endorsement in IVF. The ensuing mechanistic and clinical study is based on this methodological approach. Table 1 provides a concise summary of the primary clinical characteristics, research methodologies, and reproductive outcomes of the studies included in this narrative review.

Table 1 presents the main clinical studies examining G-CSF as an adjunct for endometrial preparation in in vitro fertilisation. Research is categorised based on clinical indication and endometrial characteristics. A systematic overview of the current clinical data includes details on research design, patient demographics, G-CSF delivery route and timing, cycle type, and key reproductive outcomes.

## 3. Biological Basis of Granulocyte Colony-Stimulating Factor in the Endometrium

### 3.1. Expression of Granulocyte Colony-Stimulating Factor and Its Receptor in Endometrial Tissue

In the human endometrium, granulocyte G-CSF and its receptor establish an intrinsic cytokine signalling axis that extends beyond haematopoietic regulation and integrates into the molecular framework governing endometrial receptivity [31]. G-CSF is expressed in the endometrial epithelium and stroma, exhibiting a spatial distribution that promotes coordinated paracrine and autocrine signalling during the peri-implantation period, as evidenced by transcriptomic profiling, immunohistochemistry, and in situ hybridisation. As the endometrium transitions into the secretory phase, stromal expression rises, suggesting stage-specific functional responsibilities [32]. Luminal and glandular epithelial cells seem to be the primary source of G-CSF.

The expression of the G-CSFR is more restricted and physiologically informative. The principal cellular compartments responsible for decidual transformation, immunological regulation, and vascular adaptation are stromal fibroblast-like cells, decidualized stromal cells, and endothelial cells of the endometrial microvasculature, where G-CSFR is mostly located [33]. G-CSF functions as a mediator of epithelial stromal vascular crosstalk rather than a generalised proliferative signal due to its compartmentalised receptor distribution, indicating that epithelial-derived G-CSF primarily exerts paracrine effects on stromal and endothelial targets [34].

Significantly, the expression of G-CSF and G-CSFR is dynamically modulated throughout the menstrual cycle. The window of implantation and progesterone dominance aligns with the mid-luteal phase, during which expression reaches its zenith [35]. This temporal regulation indicates a functional relationship between cytokine-responsive transcriptional activation and progesterone-dependent chromatin remodelling. In stromal cells, progesterone signalling establishes a permissive epigenetic milieu characterised by increased chromatin accessibility at decidual gene loci [7]. This enables cytokine-activated transcription factors such as STAT3 to efficiently interact downstream of G-CSFR signalling. In this context, G-CSF response seems to be contingent upon prior hormonal priming rather than operating independently of endocrine signals [36].

G-CSFR has no intrinsic kinase activity and belongs to the class I cytokine receptor family at the molecular level. Following ligand interaction that induces receptor homodimerization, associated Janus kinases, namely JAK2, are recruited and activated [37]. The cytoplasmic tail of G-CSFR contains conserved tyrosine residues that are phosphorylated by active JAK2, creating docking sites for STAT family transcription factors. STAT3 serves as the primary downstream effector in endometrial cells due to its documented roles in decidualisation, immunological tolerance, and vascular stability [38]. A direct molecular link between external cytokine availability and intracellular transcriptional regulation of receptivity-associated programs is facilitated by a fully operational G-CSFR–JAK2–STAT3 signalling module in endometrial stromal and endothelial cells.

In addition to conventional signalling, recent research demonstrates that transcriptional and epigenetic contexts influence G-CSFR signalling efficacy in the endometrium. Chromatin accessibility at loci associated with implantation is controlled by STAT3’s interactions with chromatin-modifying complexes, including histone acetyltransferases and methyltransferases [39]. Consequently, instead of initiating whole new gene expression programs, G-CSF-induced STAT3 activation may serve as a transcriptional amplifier in a progesterone-primed endometrium, reducing the activation threshold for decidual and immune-regulatory genes. This process is particularly significant in endometrial tissue that is morphologically apparent but functionally constrained [14].

Notably, G-CSFR expression seems to persist in pathological endometrial states, such as treatment-resistant thin endometrium, characterised by diminished oestrogen response. This preservation indicates that, even when conventional estrogen-driven proliferative pathways are diminished or epigenetically constrained, cytokine-mediated signalling pathways remain available. This differentiation is essential from a molecular perspective: Intact G-CSFR signalling provides an alternative molecular pathway for modulating stromal competence, immunological tolerance, and vascular integrity, but oestrogen resistance limits further proliferative growth.

The expression of G-CSFR in endothelial cells introduces an additional dimension of biological importance. G-CSF signalling has been associated with enhanced survival signalling, resistance to oxidative and inflammatory stress, and maintenance of intercellular junctional integrity in endothelial cells. These effects are expected to encourage regulated perfusion and endothelial quiescence instead of heightened angiogenic activity inside the endometrium, hence facilitating microvascular stabilisation throughout the peri-implantation phase. Alongside stromal decidualisation and immunological adaptation, vascular stabilisation is becoming seen as an essential prerequisite for successful implantation.

### 3.2. G-CSF-Activated Signaling Pathways in Endometrial Cells

The engagement of granulocyte colony-stimulating factor with its receptor activates a systematically structured intracellular signalling network that incorporates hormonal priming, local tissue environment, and cytokine sensitivity [40]. This signalling in endometrial cells shows compartment-specific regulation, reflecting the unique functional functions of stromal, immunological, and endothelial populations after implantation, rather than exhibiting a linear or uniform pattern across cell types. The activation of the JAK-STAT pathway is essential to this network. It serves as the primary transcriptional conduit linking the presence of extracellular G-CSF to the reconfiguration of endometrial function [41].

Upon ligand binding to G-CSFR, a conformational shift occurs, resulting in homodimer formation that facilitates the recruitment and activation of JAK2. Upon activation, JAK2 phosphorylates conserved tyrosine residues within the receptor’s intracellular domain, therefore generating docking sites for STAT family members [37]. STAT3 serves as the primary downstream effector in the endometrium, aligning with its established functions in vascular adaptation, immunological tolerance, and decidualisation. The phosphorylation and nuclear translocation of STAT3 are critical biological checkpoints, since this transcription factor regulates gene expression in response to progesterone and cytokines [42]. In endometrial stromal cells, progesterone receptor signalling and STAT3 activation interact directly. STAT3 interaction facilitates transcription, essential for gene expression, whereas progesterone modifies the stromal chromatin architecture, enhancing accessibility to decidual gene loci. The cooperative relationship is especially evident at the HOXA10 promoter, where STAT3 binding enhances the transcription of a key regulator of endometrial receptivity [42]. HOXA10 regulates a network of genes implicated in modifying the extracellular matrix, synthesising integrins, producing cytokines, and facilitating communication between epithelial and stromal cells. G-CSF signalling via this pathway does not induce nonspecific proliferation; rather, it reinforces decidual identity [43].

G-CSF stimulates the PI3K–AKT pathway in conjunction with JAK–STAT signalling, which is essential for cellular survival, metabolic adaptability, and resistance to apoptotic stress. The PI3K-AKT signalling pathway in decidualizing stromal cells promotes lipid metabolism, glucose absorption, and mitochondrial integrity, all of which are crucial for maintaining the energy-intensive decidual transformation process [44]. In the peri-implantation period, AKT-mediated suppression of pro-apoptotic proteins bolsters the stability of stromal cell populations, averting premature cell loss that may jeopardise tissue integrity. These signals that support cellular survival collaborate to enhance stromal functional competence by augmenting STAT3-mediated transcriptional processes [45]. The MAPK signalling cascade is an additional mechanism by which G-CSF regulates cells and facilitates alterations in their cytoskeleton. Actin dynamics and focal adhesion turnover, crucial for stromal cell spreading and matrix contact during decidualisation, are linked to ERK1/2 activation. The MAPK signalling pathway, in isolation, is insufficient to induce decidual differentiation; however, its interaction with the STAT3 and PI3K pathways may simultaneously elicit structural and transcriptional alterations. This convergence highlights G-CSF’s function as a systems-level modulator rather than just a unique route activator [46].

G-CSF-induced signalling affects the epigenetic regulation of endometrial cells, in conjunction with transcriptional activation. Studies indicate that STAT3 interacts with chromatin-modifying complexes, including histone acetyltransferases and methyltransferases, to promote permissive chromatin configurations at loci linked to implantation [47]. In progesterone-primed but functionally compromised endometrium, such epigenetic remodelling may reduce the threshold for the activation of decidual genes. This process elucidates how G-CSF might alter the functionality of endometrial tissue, rendering it resistant to subsequent estrogen-mediated proliferation [48]. The effects of G-CSF signalling are further defined by the specialisation of cell types. Activation of the JAK2–STAT3 and PI3K-AKT pathways in endothelial cells enhances nitric oxide availability, stabilises intercellular junctions, and promotes cell survival. These actions improve microvascular integrity and control perfusion, rather than provoking excessive angiogenic sprouting [49]. G-CSF signalling indirectly modifies cytokine networks in the immune system, reducing pro-inflammatory pathways and promoting tolerogenic signalling that improves macrophage polarisation and regulatory T-cell proliferation. The immunomodulatory effects are amplified by paracrine signalling from stromal and epithelial cells, even if immune cells in the endometrium may not consistently display increased levels of G-CSFR [50]. Negative feedback and cross-regulatory mechanisms that impose time constraints on receptivity significantly influence G-CSF-activated signalling pathways. Suppressors of cytokine signalling proteins, particularly SOCS3, which are activated after the activation of STAT3, attenuate signalling intensity. This intrinsic feedback prevents prolonged or excessive route activation from disrupting the meticulously controlled implantation window. These regulatory mechanisms support the notion that G-CSF signalling is constrained by physiological and contextual factors [51].

### 3.3. Effects of Granulocyte Colony-Stimulating Factor on Stromal Decidualization and Tissue Remodeling

Decidualisation of endometrial stromal cells converts fibroblast-like stromal cells into specialised decidual cells that enable trophoblast invasion, promote immunological tolerance, and facilitate vascular remodelling, acting as a vital biological checkpoint for implantation. This alteration is induced by progesterone-dependent transcriptional mechanisms [52]. However, additional cytokine-mediated signals are requisite for complete functional competence. Granulocyte colony-stimulating factor amplifies stromal response in a hormonally primed endometrium by acting as a modulatory signal that strengthens decidual differentiation rather than beginning it afresh [53].

G-CSF mostly stimulates the STAT3–HOXA10 pathway to promote stromal decidualisation at the molecular level. The phosphorylation of STAT3 facilitates its translocation into the nucleus, enabling direct binding to regulatory areas of genes associated in decidualisation, such as HOXA10 and FOXO1 [53]. It facilitates the organisation of the extracellular matrix and the release of cytokines by downstream effectors. HOXA10 primarily governs integrin expression, stromal-epithelial communication, and matrix remodelling, establishing it as the principal coordinator of decidual competence. G-CSF signalling may promote functional differentiation and contribute to the stabilisation of the decidual phenotype, particularly in hormonally primed endometrial tissue. [54].

The stimulation of PI3K-AKT signalling by G-CSF enhances the survival of stromal cells and their metabolic adaptability. Decidualisation is an energy-demanding process necessitating increased glucose absorption, lipid metabolism, and mitochondrial alterations to maintain secretory and structural functions [55]. During the peri-implantation period, AKT activity prevents early stromal cell loss by maintaining mitochondrial integrity and inhibiting apoptotic pathways. These survival signals are crucial in a thin or chronically unresponsive endometrium, where cellular quantity and regenerative capacity may be restricted [56]. G-CSF alters the extracellular matrix and the cytoskeleton via MAPK-dependent signalling. Activation of ERK1/2 facilitates actin reorganisation, focal adhesion turnover, and cell–matrix interactions. All of these factors are crucial for stromal cell dissemination and decidual architecture. These structural modifications facilitate the formation of a flexible stromal matrix that permits invasion while preserving tissue integrity [57]. This kind of remodelling must be meticulously regulated and coincide with transcriptional differentiation to prevent excessive fibrosis or disordered matrix deposition.

G-CSF may influence the behaviour of progenitor-like cell populations in the endometrium, as well as its direct impact on differentiated stromal cells. Data from several tissues suggest that G-CSF may mobilise, attract, or activate progenitor populations involved in tissue repair, notwithstanding the uncertainty about the nature and function of endometrial progenitor cells [53]. This pathway may facilitate regeneration processes in the endometrium after prolonged inflammatory stress, surgical damage, or repeated hormonal stimulation. The specific effectiveness of G-CSF in severe thin endometrium may be clarified by its regenerative support, perhaps facilitating the restoration of functional competency in impaired or resistive endometrial tissue [19].

During decidualisation, careful regulation of extracellular proteases and inflammatory mediators is essential for tissue remodelling. G-CSF signalling regulates the stromal cytokine milieu, preventing excessive inflammation and facilitating the production of factors that enhance immune system tolerance and matrix remodelling [58]. This balanced cytokine profile prevents premature degradation of the stromal tissue and facilitates regulated invasion by the trophoblast. G-CSF facilitates the establishment of a decidual environment that is both permissive and restrictive by maintaining the equilibrium between inflammation and remodelling. The effects of G-CSF on stromal decidualisation are temporally and contextually constrained [59]. Progesterone priming is required to prepare the chromatin environment for STAT3-mediated transcriptional activation. Insufficient hormonal conditioning or administration of G-CSF outside the implantation window may hinder the activation of these pathways. The need for exact timing indicates that administering G-CSF to IVF patients routinely or without a particular justification is ineffective.

### 3.4. Immunomodulatory Effects of Granulocyte Colony-Stimulating Factor in the Endometrial Microenvironment

Successful embryo implantation requires an optimally balanced immunological milieu, allowing the semi-allogeneic embryo to be identified and integrated without eliciting excessive inflammation [16]. The responsive endometrium experiences active immunological reprogramming, marked by the temporal and geographical synchronisation of innate and adaptive immune cell populations, rather than a condition of immunosuppression [60]. Granulocyte colony-stimulating factor is crucial in this process since it alters immune cell activity and cytokine signalling, hence fostering tolerance, tissue remodelling, and vascular adaptability.

G-CSF largely influences immunological modulation by indirectly governing the recruitment, differentiation, and functional polarisation of immune cells at the molecular level. Stromal and epithelial cells display a vigorous response to G-CSF signalling, thereby altering the immune milieu through the release of paracrine cytokines and chemokines, despite classical immune cells in the endometrium not consistently exhibiting heightened G-CSF receptor expression [61]. G-CSF may modify immune system functionality without diminishing its efficacy, therefore maintaining immune surveillance while fostering tolerance conducive to implantation.

Regulatory T cells are essential for immunological tolerance associated with implantation. To prevent the maternal immune system from excessively assaulting the embryo during the peri-implantation phase, Treg cells must proliferate and activate [62]. G-CSF signalling has been shown to facilitate Treg development and stability by using cytokines to enhance FOXP3 expression and inhibit pro-inflammatory Th1 and Th17 pathways. This alteration enhances the endometrium’s capacity to function effectively with the immune system, facilitating embryo acceptance while maintaining responsiveness to potential dangers. A deficiency in Treg dominance has been linked to recurrent implantation failure and early pregnancy loss, underscoring the importance of G-CSF-mediated Treg regulation [63].

Macrophages represent a significant category of immune cells influenced by G-CSF signalling. Macrophages in the receptive endometrium polarise to an M2-like phenotype, which fosters angiovascular stability, produces anti-inflammatory cytokines, and modifies the extracellular matrix [64]. G-CSF indirectly facilitates this polarisation by influencing cytokine production in stromal and epithelial cells and elevating the amounts of IL-10, TGF-β, and other mediators that enhance the body’s tolerance. M2 macrophages mitigate excessive inflammatory damage while promoting regulated trophoblast invasion and tissue remodelling. The significance of this route is shown by the correlation between implantation failure and the deregulation of macrophage polarization [65].

During the first phases of pregnancy, uterine natural killer cells constitute the predominant immune cells in the decidua. They possess a distinct function that differentiates them from peripheral NK cells [66]. Rather than being detrimental to cells, uNK cells secrete growth and angiogenic substances that facilitate the remodelling of spiral arteries and the adaptation of blood vessels. Although G-CSF signalling does not seem to directly influence uNK cells, signals from stromal and macrophage cells impact the cytokine milieu that governs their functional development [67]. G-CSF may indirectly facilitate uNK cell differentiation towards a vascular-supportive phenotype by stabilising the cytokine network and augmenting immune-vascular coupling after implantation [16].

STAT3-dependent transcriptional programs that govern cytokine synthesis and immune cell communication interact with G-CSF-mediated immune regulation at the signalling level. In stromal cells, the activation of STAT3 augments the production of proteins that promote immunological tolerance while suppressing excessive pro-inflammatory signalling [68]. The activation of suppressor of cytokine signalling proteins, especially SOCS3, which provide negative feedback to terminate chronic inflammatory activation, further strengthens this transcriptional balance. These regulatory mechanisms restrict immune modulation to the period of implantation [69].

The immunomodulatory effects of G-CSF are critically reliant on context and need a well-prepared endometrial environment. Exposure to progesterone creates a fundamental tolerogenic state, which is later altered by cytokine signalling. G-CSF-induced immune modulation may be insufficient for reinstating receptivity in instances of poor hormone priming or when immune dysregulation is triggered by exogenous causes, such as inflammation linked to endometriosis. This reliance likely elucidates why various patient cohorts do not consistently exhibit uniform responses to therapy.

### 3.5. Vascular Stabilization and Microcirculatory Adaptation

For the endometrium to exhibit receptivity, the vascular network must be functionally mature, stable, and meticulously regulated. Excessive angiogenesis is unnecessary for implantation. It depends on the integrity of the endothelium, regulated perfusion, and the synchronised interplay of the stromal, immunological, and vascular components [55]. Despite sufficient endometrial thickness, microvascular dysfunction results in insufficient oxygen supply, inflammatory activation, and diminished decidual support.

Microvascular instability, rather than only vascular insufficiency, often contributes to the deterioration of the endometrium. A thin and chronically unresponsive endometrium often exhibits reduced perfusion, endothelial cell dysfunction, increased vascular permeability, and abnormal inflammatory signalling [18]. These alterations impede stromal decidualisation and immunological tolerance, while concurrently increasing the likelihood of local hypoxia and the activation of stress pathways. Estrogen-induced proliferation does not improve vascular disease, underscoring the need for therapies that precisely target endothelial function [16].

The available data suggests that granulocyte colony-stimulating factor may influence endometrial receptivity by stabilising and enhancing the microvascular environment, rather than primarily facilitating angiogenesis. When ligands activate endothelial cells in the endometrium, they produce functional G-CSF receptors and initiate pathways that enhance their survival and stress response [70]. These signals assist endothelial cells in maintaining viability and safeguarding them from oxidative or inflammatory injury during the peri-implantation period [71].

At the molecular level, G-CSF activates PI3K-AKT and JAK2-STAT3 signalling in endothelial cells. STAT3 promotes the quiescence of endothelial cells and inhibits the transcription of pro-inflammatory genes [72]. AKT signalling facilitates vasodilation and enhances microcirculatory efficiency. It also enhances the activity of nitric oxide synthase and the availability of nitric oxide. Collectively, these mechanisms facilitate functional perfusion while minimising excessive angiogenic remodelling [73]. G-CSF also influences the stability of endothelial connections. The activation of survival signalling maintains the integrity of adherens and tight junctions by reducing vascular permeability and preventing the extravasation of inflammatory cells from the bloodstream. This stability safeguards the stromal compartment from stress responses induced by hypoxia by limiting the localised amplification of cytokines [74]. Chronic low-grade inflammation damages the vascular barriers in diseased endometrium, making it crucial to reduce endothelial permeability.

G-CSF-induced vascular signalling interacts with hypoxia-responsive pathways critical for implantation damage. Hypoxia-inducible factors, which inhibit decidual gene expression and initiate inflammatory signalling, are perpetually activated in dysfunctional endometrium due to inadequate perfusion [75]. G-CSF may diminish pathological hypoxia signalling by enhancing microcirculatory flow and endothelial integrity, so fostering a metabolic state conducive to decidual transformation. G-CSF influences the implantation niche by facilitating communication between immune and endothelial cells [76]. The stabilised endothelium promotes the development of uterine natural killer cells into a vascular-supportive phenotype while limiting the recruitment of unwanted immune cells. In the first stages after implantation, this synchronised modulation preserves immunovascular balance and improves spiral artery remodelling [77]. Clinical findings validate this mechanistic theory. Research demonstrates improvements in endometrial blood flow metrics, vascularization flow index, and endometrial volume after intrauterine G-CSF treatment. These alterations signify functional optimisation rather than structural enlargement, since they transpire without excessive angiogenesis. Patients with pre-existing microvascular dysfunction have the most pronounced vascular response due to the context-dependent therapeutic effectiveness of G-CSF [78].

## 4. Clinical Application of Granulocyte Colony-Stimulating Factor in In Vitro Fertilization

### 4.1. Granulocyte Colony-Stimulating Factor in Thin Endometrium

A thin endometrium is not only a deficiency in quantity. It is a distinct clinical condition. In several afflicted cases, the failure to attain appropriate thickness is ascribed not to inadequate oestrogen exposure, but to impaired stromal response, vascular dysfunction, and poor tissue remodelling [18]. Enhancing hormonal stimulation may exacerbate inflammatory and vascular stress and often fails to restore functional ability. The need to maintain cycles that would otherwise be voided led to the therapeutic use of G-CSF in thin endometrium. In women with treatment-resistant thin endometrium, intrauterine infusion of G-CSF has consistently shown an increase in endometrial thickness [79]. However, the therapeutic outcomes reported cannot be exclusively ascribed to thickness enhancement. Significantly, even with optimal endocrine support, G-CSF seems capable of reinstating transfer eligibility in instances when prior cycles have been unsuccessful.

This response corresponds with the molecular effects of G-CSF on immunological regulation, vascular stability, and stromal preservation from a mechanistic viewpoint. Prevalent baseline diseases in thin endometrium include elevated hypoxia signalling, endothelial instability, and insufficient microcirculation [80]. G-CSF enhances endothelial function and microvascular perfusion, hence augmenting stromal metabolic support and response to progesterone. Based on indirect molecular and clinical observations, this may aid in the restoration of certain functional aspects of decidual competence. The pregnancy results in persons with a thin endometrium are variable. Certain studies reveal increased rates of implantation and pregnancy, while others show improvements in thickness without a proportional increase in live births. This discrepancy is likely attributable to variations in the extent of damage remaining in the endometrium at the time of medication administration and the initial condition of the tissue [81]. Individuals demonstrating functional impairment, rather than permanent structural damage, seem to get the most advantage from G-CSF.

Crucially, G-CSF does not induce abnormal or uncontrolled angiogenesis. Its actions are confined to stabilising tissue structure and enhancing its function. This differentiates G-CSF from estrogen-based methods and affirms its function as a conditioning agent rather than a proliferative treatment. Generally, G-CSF may assist some individuals with a thin endometrium by addressing issues related to blood vessels and stroma. The endometrial phenotype and previous response to conventional preparation should determine its use, which must be specific rather than standard.

### 4.2. Granulocyte Colony-Stimulating Factor in Recurrent Implantation Failure

Recurrent implantation failure is a complex clinical syndrome defined by the lack of implantation after successive transfers of embryos with viable developmental potential. RIF is often identified by the absence of noticeable morphological or hormonal irregularities, unlike diseases characterised by clear endometrial deficiency [82]. The lack of noticeable anomalies complicates therapy and suggests that modest changes in endometrial function, rather than major physical issues, are the cause of implantation failure in these patients. Endometrial asynchrony is a fundamental attribute of RIF [83]. If the molecular and cellular processes required for implantation occur outside the optimal time frame, a discrepancy may arise between endometrial receptivity and embryonic signalling. Minor temporal alterations may disrupt the sequence of adhesion, invasion, and decidual support. In this scenario, G-CSF may affect the timing and synchronisation of endometrial responses during the peri-implantation period, although it is not expected to induce substantial morphological changes [19].

A recurring issue in the pathogenesis of RIF is immunological dysregulation. A significant number of patients lack a tolerant, implantation-permissive condition due to dysfunction in their immune systems. Rather than just experiencing excessive inflammation, they have aberrant immune activation patterns [84]. This includes impaired immune-vascular communication and altered regulatory immune cell dynamics. G-CSF may be advantageous in this setting by enhancing immune recalibration rather than suppression, so permitting more effective physiological immune adaptation during early implantation [85]. The clinical results documented in RIF studies demonstrate this functional mode of action. The thickness or shape of the endometrium has not changed in conjunction with improvements in implantation or early pregnancy rates [86]. The data demonstrate that G-CSF influences qualitative aspects of endometrial function not captured by traditional clinical evaluations. The complexity of recurrent implantation failure is highlighted by the inconsistent live birth rates seen in studies, indicating that only certain biological subtypes may demonstrate a response. The manner of G-CSF administration introduces more variability [82]. Systemic injection may indirectly affect the uterine environment by altering broad immunological networks and the migration of peripheral immune cells. In contrast, local intrauterine delivery may particularly affect endometrial signalling pathways. Diverse outcomes stem mostly from diversity in patient selection, scheduling, and study design, underscoring the absence of a standardised treatment protocol [87].

RIF is not a singular disease entity, which is significant. G-CSF is unlikely to be beneficial when implantation failure primarily results from embryonic factors, genetic abnormalities, or significant uterine pathology [83]. In other instances, the main mechanism may be functional endometrial dysregulation. The variable results seen in clinical trials and meta-analyses are likely due to the failure to distinguish between these variables [88]. The aggregated data suggests that G-CSF may benefit a specific group of RIF patients with functional endometrial dysregulation, as opposed to being limited by structural or developmental factors. Therefore, its role should be considered hypothesis-driven and selected [89]. The future clinical significance will depend on improved phenotyping techniques that may identify individuals for whom the main barrier to implantation is not tissue structure, but rather immunological and temporal endometrial adjustments.

### 4.3. Use of G-CSF in Unselected IVF Populations and Reasons for Neutral Outcomes

Unselected IVF populations represent a biologically diverse cohort wherein implantation failure arises from various factors, often non-endometrial in nature, as opposed to phenotypes defined by evident endometrial dysfunction [90]. Under these circumstances, the molecular programs that regulate decidualisation, immune adaptation, and vascular stabilisation operate within physiological limits, and baseline endometrial receptivity frequently remains intact. This baseline competency greatly changes the expected effects of additional treatments like G-CSF. Clinical trials evaluating G-CSF in unselected IVF cycles consistently report neutral effects on implantation and pregnancy outcomes. These results indicate the absence of a targetable pathological substrate rather than biological inefficacy itself [15]. When the stromal, immune, and vascular pathways are already in sync, adding more cytokine stimulation does not help. In this case, G-CSF signalling might just make processes that are already working close to their full potential even stronger.

At the molecular level, endometrial signalling pathways show saturation kinetics. Both cytokine-mediated immune adaptation and progesterone-driven decidualisation attain functional plateaus, beyond which further activation yields diminishing returns [91]. Consequently, it is improbable that the administration of exogenous G-CSF will modify transcriptional or cellular programs in a manner that holds clinical significance in an endometrium devoid of underlying dysfunction. The absence of response observed in commonly selected IVF cohorts can be mechanistically elucidated by this ceiling effect [15]. Timing-related factors also have an effect on neutral results. In unselected populations, implantation failure is often attributed to chromosomal abnormalities, stochastic developmental events, or embryonic competence, rather than endometrial preparedness. In these cases, even carefully timed changes to the endometrial environment cannot make up for problems that happen earlier in the process. G-CSF’s inability to traverse barriers originating from embryos underscores the necessity of aligning therapeutic strategies with the prevailing limiting factor [24].

Additionally, using immunomodulatory drugs carelessly can throw off delicate physiological processes. In a functionally normal endometrium, unnecessary immune or vascular modulation may generate biological noise rather than confer benefits, despite the general tolerance of G-CSF. This factor substantiates the rationale for restraint in populations where endometrial pathology is not distinctly apparent [12]. The disparities in outcomes between selected and unselected populations underscore a fundamental principle of reproductive medicine: endometrial interventions are effective solely when aligned with observable endometrial dysfunction. G-CSF exemplifies this concept. Instead of being a universal enhancer of implantation, its effectiveness depends on the situation, which is what makes it a conditioning signal [82]. When viewed collectively, the absence of benefit in unselected IVF populations should not be interpreted as evidence that G-CSF lacks biological significance. Instead, it stresses how important it is to use precision-based applications. Clinical trials can make it harder to see signals of efficacy and may wrongly label targeted therapies as ineffective if they do not use phenotypic stratification [92]. Consequently, enhanced patient selection should be prioritised over broader application in forthcoming research. Table 2 provides a summary of a phenotype-based synthesis about the responses of several IVF populations to granulocyte colony-stimulating factor.

Table 2 delineates the clinical effects of G-CSF in in vitro fertilisation according to their phenotypes. Thin endometrium, recurrent implantation failure, and unselected IVF populations are used to summarise variances in biological goals, response consistency, and impacts on reproductive outcomes. The table elucidates a detailed grasp of G-CSF’s use in medicine and emphasises that its efficacy is contingent upon the context.

## 5. Discussion

This narrative review evaluates granulocyte colony-stimulating factor as an additional approach for endometrial preparation in in vitro fertilisation, including both mechanistic and clinical information. The research sought to clarify the substantial variation in reported results by simultaneously assessing studies on thin endometrium, recurrent implantation failure, and unselected IVF populations, rather than just listing positive and negative trials. This study aimed to discover consistent molecular characteristics that clarify clinical response by merging current randomised trials and meta-analyses with previous proof-of-concept research. The comparison method used here enhances the understanding of G-CSF effectiveness via endometrial phenotype, degree of functional impairment, and biological timing, instead of depending on specific outcome measurements.

The current synthesis sought to resolve contradictory results in the literature by moving beyond binary interpretations of effectiveness. This discussion examines the physiologic parameters that enable G-CSF to alter processes critical for implantation, rather than assessing its efficacy. The integration of mechanistic insights with phenotype-specific clinical data provides a paradigm for comprehending the differing results of similar therapies across diverse patient groups. This concept designates G-CSF as a context-specific tool for endometrial conditioning, with its effectiveness dependent on the existence of a treatable disease, rather than as a general supplement to IVF.

Tehraninejad et al., Kunicki et al., and Xu et al. assert that granulocyte colony-stimulating factor consistently thickens the endometrium in women with unresponsive thin endometrium [20,22,92]. These studies together provide convergent evidence that the endometrium maintains a certain level of biological reactivity in the absence of regular hormonal stimulation. Nonetheless, due to variations in implantation, clinical pregnancy, and live birth rates between cohorts, Kunicki et al. and Miralaei et al. illustrate that this morphological response does not uniformly result in enhanced reproductive outcomes [22,24]. Miralaei et al. also observe that a considerable proportion of patients remain ineligible for transfer after receiving numerous doses of G-CSF [24]. This indicates that the restoration of thickness alone is insufficient to address more significant functional or structural issues. Zhang et al. substantiate this view by demonstrating that G-CSF enhances endometrial thickness and cumulative pregnancy rates in patients with significant structural impairment, such as Asherman syndrome, without preventing adhesion recurrence [53]. This illustrates the distinction between functional augmentation and genuine tissue regeneration.

Xu et al., Banerjee et al., and Kunicki et al. contribute to this narrative by demonstrating that postponing embryo transfer to frozen cycles may enhance the perceived efficacy of G-CSF for thin endometrium [22,93,94]. Xu et al. indicate that greater implantation and pregnancy rates occur after the cancellation and subsequent frozen embryo transfer, suggesting that temporal separation from ovarian stimulation improves the effectiveness of G-CSF’s biological effects [93]. Banerjee et al. validate these results, demonstrating that in some cases, systemic subcutaneous G-CSF may provide physiologic effects similar to those attained by intrauterine treatment [94]. This suggests that the delivery strategy may affect the severity and distribution of endometrial conditioning. Miralaei et al. warn that permanent stromal or vascular damage may impede receptivity, even under favourable cycle circumstances, hence increasing variability within the thin endometrium phenotype [24].

Eftekhar et al., Davari-Tanha et al., and Aleyasin et al. together refine the interpretative framework for analysing recurrent implantation failure, consistently reporting improvements in implantation or early pregnancy outcomes without significant changes in endometrial thickness [25,26,27]. Davari-Tanha et al. indicate increased rates of chemical pregnancy and implantation without long-term benefits for live births, whereas Eftekhar et al. show improved clinical pregnancy rates in RIF patients after intrauterine G-CSF treatment [25,26]. Aleyasin et al. expand upon these findings by demonstrating that systemic subcutaneous G-CSF significantly enhances implantation and clinical pregnancy rates, even when controlling for other variables [27]. This suggests that immunological or systemic variables, in addition to local morphology, may affect effectiveness. These data suggest a functional mechanism of action in this group, differentiating RIF from thin endometrium.

Barad et al., Jain et al., and Kamath et al. provide a crucial counterargument by consistently showing neutral results in unselected IVF cohorts [28,29,95]. In a rigorously structured randomised controlled study, Barad et al. demonstrate no improvement in endometrial thickness, implantation, or clinical pregnancy, whereas Jain et al. confirm the lack of advantage across several reproductive outcomes, including live birth [28,29]. Kamath et al. further show that pooled effect sizes decrease and the confidence of the evidence remains low when studies include widely chosen IVF cohorts, as evidenced by systematic review and meta-analysis [95]. The data suggest that G-CSF does not improve implantation when endometrial receptivity pathways operate within normal physiological parameters, and the lack of disease prevents therapeutic intervention.

Hou et al., Fu et al., and Kamath et al. elucidate these contradictory findings, indicating that the primary cause of clinical heterogeneity is in the selection criteria of patients rather than variations in biological effects [89,92,95]. Hou et al. demonstrate via subgroup analysis that the advantages of G-CSF are mostly seen in women with a thin endometrium or recurrent implantation failure, irrespective of the administration method or cycle type [89]. Fu et al. validate neutral results in generic IVF cohorts, while simultaneously documenting improved clinical pregnancy and implantation rates in these particular individuals [92]. Kamath et al. assert that biological variability and methodological constraints, rather than a lack of impact among responsive subgroups, account for the uneven live birth advantage and the low confidence of the findings [95].

Arefi et al. and long-term cohort studies elucidate this view, indicating that G-CSF is advantageous only for certain subgroups of patients with unexplained or immune-related dysfunction in recurrent implantation failure [96]. Cohort data reveal varying responses based on baseline immunological and endometrial functional conditions. Arefi et al. demonstrate enhanced results in cases of unexplained RIF [96]. These data highlight that a primary shortcoming of existing studies, and a significant factor in the diminished treatment outcomes, is the failure to profile individuals at a biological level. A comparative study of research indicates that the clinical translation of G-CSF’s consistent biological effects is contingent upon the existence, severity, and reversibility of endometrial dysfunction. While neutral results in unselected IVF populations suggest the need for an underlying disease substrate, sustained gains in the thickness of thin endometrium contrast with thickness-independent implantation improvements shown in recurrent implantation failure. These converging trends support a specific interpretation of G-CSF as a focused endometrial conditioning approach rather than a generic adjunct to IVF, hence resolving any inconsistencies in the literature. Table 3 illustrates the correlation between endometrial abnormalities, proposed mechanisms of action, and clinical outcomes from various studies.

Table 3 encompasses the predominant biological processes, endometrial pathologies, and clinical outcomes from IVF studies associated with G-CSF. The table illustrates the dependence of G-CSF efficacy on contextual factors and the influence of the existence, severity, and reversibility of endometrial dysfunction on its effectiveness.

## 6. Limitations of Current Evidence and Future Directions

A burgeoning corpus of clinical research assesses granulocyte colony-stimulating factor in assisted reproduction; nonetheless, the validity and relevance of current results are limited by many considerations. The significant variability in patient selection, including thin endometrium, recurrent implantation failure, unselected IVF populations, and mixed cohorts often examined within a unified treatment framework, represents a substantial study restriction. The variable outcomes seen in trials and meta-analyses are likely due to the lack of phenotypic stratification, which conceals genuine biological signals.

A significant issue is that the research designs and outcome measurements are not consistently uniform. Numerous studies examine intermediate goals like as endometrial thickness, biochemical pregnancy, or implantation rate, rather than live birth and neonatal outcomes, which are either inadequately powered or inconsistently reported. This disparity complicates the comprehension of clinical importance, particularly when initial implantation benefits do not result in sustained pregnancy. Furthermore, variations in the kind of cycle, timing of administration, dosage, and method of delivery complicate direct comparisons across trials. Limited sample numbers, poor blinding, and insufficient correction for confounding variables across many trials constitute further methodological constraints. The robustness of pooled estimates is compromised by insufficient power and inconsistent definitions of recurrent implantation failure, even within randomised controlled settings. Meta-analyses are beneficial; nonetheless, they possess some limitations. For instance, they often combine groups of individuals with biological differences, resulting in diminished effect sizes and less evidential certainty.

The lack of verified biomarkers is a significant issue from a biological perspective. Many of the criteria used by physicians for decision-making are predicated on visual assessments or historical data, neglecting the functional efficacy of the endometrium. The injection of G-CSF is still empirical rather than targeted since there are no molecular, immunological, or vascular indicators to identify response phenotypes. This constraint may result in superfluous therapies for patients unlikely to gain an advantage, while reducing therapeutic effectiveness. Future research should prioritise phenotype-driven trial design. Stratification using vascular measures, immunological signatures, or endometrial functional profiles may identify patient cohorts most likely to react to G-CSF. Integrating transcriptomic, proteomic, or immunophenotypic data may facilitate the development of biomarkers capable of predicting therapy outcomes and enhancing treatment recommendations. Precision endometrial conditioning would replace the indiscriminate use of adjuvants in the field.

Additional study is required to improve timing, dose, and delivery method. Comparative studies investigating intrauterine vs. systemic administration within well-defined phenotypes may clarify the advantages of localised or systemic immune regulation in certain circumstances. Standardised reporting of live birth, obstetric, and neonatal outcomes is crucial for evaluating long-term safety and genuine therapeutic benefit. Subsequently, future studies need to include mechanistic objectives in conjunction with clinical results. Establishing a connection between endometrial molecular alterations and successful reproduction will improve causal inference and integrate experimental biology with clinical practice. Integrative investigations are necessary to completely clarify the function of G-CSF in the advancing field of personalised reproductive medicine.

## 7. Conclusions

Granulocyte colony-stimulating factor is a therapeutically targeted and physiologically feasible adjuvant for endometrial preparation in in vitro fertilisation. The comprehensive data provided indicates that G-CSF acts as a context-dependent regulator of endometrial functional competence, rather than a universal enhancer of implantation. An underlying, physiologically changeable endometrial malfunction has a greater impact on clinical relevance than the intervention itself. Identical patterns consistently emerge throughout all research. Pregnancy results are inconsistent and depend on the reversibility of tissue pathology; yet, individuals with treatment-resistant thin endometrium exhibit reliable enhancements in endometrial thickness. The advantages of repeated implantation failure are mostly apparent in early reproductive results and seem to be largely unaffected by morphological alterations, indicating a functional reorganisation of the endometrial milieu. In contrast, unselected IVF groups regularly demonstrate neutral results, indicating that G-CSF provides few advantages without a unique pathophysiological basis.

These findings explain a significant portion of the perceived discrepancies in the literature. Diverse clinical results arise from variations in patient phenotype, timing, and therapy environment, rather than from dissimilar biological effects. G-CSF has little to no efficacy in practical applications. However, when used for a particular endometrial malfunction, it may enhance the likelihood of implantation and prepare the body for optimal function. To progress in this subject, it is essential to shift from dependence on experiential techniques to precision-oriented approaches. Establishing the conclusive clinical significance of G-CSF necessitates the identification of predictive biomarkers, enhancement of patient categorisation, and integration of functional endometrial evaluation into trial design. G-CSF should be considered a particular endometrial conditioning strategy rather than a conventional adjunct in assisted reproduction until these methods are used.

## Figures and Tables

**Table 1:** Clinical studies evaluating granulocyte colony-stimulating factor for endometrial preparation in in vitro fertilization.

Author (Year)	Study Design	Patient Population/Indication	Cycle Type	Route of G-CSF Administration	Timing of Administration	Primary Endpoint(s)	Main Findings
Tehraninejad et al. (2015), [20]	Non-randomized clinical trial	Thin endometrium with previous cycle cancellation	Fresh	Intrauterine	Day of oocyte retrieval or 5 days before embryo transfer	Endometrial thickness, clinical pregnancy	Significant increase in endometrial thickness; limited clinical pregnancy rate
Kunicki et al. (2014), [21]	Prospective cohort	Treatment-resistant thin endometrium	Fresh	Intrauterine	Day of ovulation trigger	Endometrial thickness, clinical pregnancy	Significant increase in thickness; modest pregnancy outcomes
Kunicki et al. (2017), [22]	Controlled cohort	Thin endometrium in frozen embryo transfer cycles	Frozen	Intrauterine	During endometrial preparation	Endometrial thickness, clinical pregnancy, live birth	Thickness improved; no significant improvement in pregnancy or live birth
Xu et al. (2015), [23]	Comparative cohort	Thin endometrium after cancelled embryo transfer cycles	Frozen	Intrauterine	During hormonal preparation	Implantation rate, clinical pregnancy	Higher implantation and pregnancy rates compared with controls
Miralaei et al. (2019), [24]	Case series	Treatment-resistant thin endometrium	Frozen	Intrauterine	During endometrial preparation	Endometrial thickness, cycle cancellation	Thickness increased; high rate of cycle cancellation persisted
Eftekhar et al. (2016), [25]	Randomized controlled trial	Recurrent implantation failure	Fresh	Intrauterine	Day of ovum pick-up	Clinical pregnancy	Significant improvement in clinical pregnancy rate
Davari-Tanha et al. (2017), [26]	Double-blind randomized controlled trial	Recurrent implantation failure	Fresh/Frozen	Intrauterine	Oocyte retrieval or progesterone initiation	Implantation rate, pregnancy outcomes	Increased implantation and chemical pregnancy; no live birth benefit
Aleyasin et al. (2016), [27]	Multicenter randomized controlled trial	Repeated IVF failure	Fresh	Subcutaneous	Before embryo implantation	Implantation rate, clinical pregnancy	Significant improvement in implantation and clinical pregnancy rates
Barad et al. (2014), [28]	Double-blind placebo-controlled randomized trial	Unselected IVF population	Fresh	Intrauterine	Day of ovulation trigger	Endometrial thickness, implantation, and clinical pregnancy	No significant effect of G-CSF on any outcome
Jain et al. (2018), [29]	Double-blind placebo-controlled randomized trial	Unselected IVF population	Fresh	Intrauterine	Day of ovulation trigger	Clinical pregnancy, live birth	No improvement in pregnancy or live birth rates
Zhang et al. (2022), [30]	Double-blind randomized controlled trial	Asherman syndrome after hysteroscopic adhesiolysis	Mixed	Intrauterine	Postoperative (7 days after surgery)	Endometrial thickness, cumulative pregnancy, and live birth	Improved thickness and cumulative pregnancy; no reduction in adhesion recurrence

**Table 2:** Phenotype-based comparison of clinical response to granulocyte colony-stimulating factor in in vitro fertilization.

Endometrial Phenotype	Dominant Pathological Feature	Primary Biological Target of G-CSF	Effect on Endometrial Thickness	Effect on Implantation Rate	Effect on Clinical Pregnancy	Effect on Live Birth	Consistency of Findings	Overall Clinical Interpretation
Thin endometrium	Impaired stromal responsiveness and microvascular dysfunction	Stromal survival, vascular stabilization	Consistent increase	Variable	Variable	Inconsistent	Moderate	Restores transfer eligibility; benefit depends on reversibility of tissue pathology
Recurrent implantation failure	Functional immune–stromal and temporal dysregulation	Immune modulation, stromal–immune synchrony	No significant change	Frequent improvement	Modest improvement	Inconsistent	Moderate to high	Functional benefit independent of morphology; selective use recommended
Unselected IVF population	Absence of dominant endometrial pathology	No clear biological target	No effect	No effect	No effect	No effect	High	No benefit; routine use not supported
Asherman syndrome/severe structural damage	Fibrosis and loss of functional endometrial tissue	Support of residual functional endometrium	Partial increase	Limited	Limited	Limited	Low	Adjunctive role only after surgical correction
Irreversible endometrial pathology	Non-modifiable tissue damage	Insufficient biological leverage	No effect	No effect	No effect	No effect	Low	G-CSF not indicated

**Table 3:** Alignment of endometrial pathology, biological mechanisms, and clinical outcomes following granulocyte colony-stimulating factor administration.

Endometrial Pathology	Predominant Functional Deficit	Principal Biological Action of G-CSF	Expected Molecular/Functional Effect	Observed Clinical Response	Strength of Clinical Evidence	Translational Implication
Treatment-resistant thin endometrium	Impaired stromal responsiveness and microvascular instability	Stromal cell survival and vascular stabilization	Improved decidual competence, enhanced perfusion	Increased endometrial thickness; variable implantation and pregnancy rates	Moderate	Useful to restore transfer eligibility in selected patients
Thin endometrium with partial reversibility	Functional stromal and vascular dysfunction	Functional endometrial conditioning	Improved receptivity without full structural normalization	Improved implantation in selected cases	Moderate	Benefit depends on the degree of residual functional tissue
Recurrent implantation failure	Immune–stromal asynchrony and impaired temporal coordination	Immune modulation and synchronization of endometrial readiness	Improved implantation signaling without morphological change	Improved implantation and early pregnancy; inconsistent live birth	Moderate	Selective use in biologically unexplained RIF
Recurrent implantation failure with systemic immune involvement	Peripheral and local immune dysregulation	Systemic immunomodulation	Improved immune tolerance and endometrial–embryo dialogue	Improved implantation and clinical pregnancy	Moderate	The systemic route may be preferable in selected RIF phenotypes
Unselected IVF population	Absence of dominant endometrial dysfunction	Minimal biological leverage	Signaling saturation	No improvement across reproductive outcomes	High	Routine use is not justified
Structural endometrial damage (e.g., Asherman syndrome)	Fibrosis and reduced functional endometrial reserve	Support of residual endometrial function	Partial recovery of receptivity without regeneration	Limited pregnancy benefit	Low	Adjunctive only after surgical correction
Irreversible endometrial pathology	Non-modifiable tissue loss	Insufficient biological response	No meaningful functional effect	No clinical benefit	Low	G-CSF not indicated

## Data Availability

No new data were created or analysed in this study. Data sharing is not applicable to this article.

## Abbreviations

The following abbreviations are used in this manuscript:

AKT	Protein kinase B
Asherman syndrome	Intrauterine adhesions syndrome
CEBPβ	CCAAT/enhancer-binding protein beta
ERK1/2	Extracellular signal-regulated kinases 1 and 2
FOXO1	Forkhead box O1
FOXP3	Forkhead box P3
G-CSF	Granulocyte colony-stimulating factor
G-CSFR	Granulocyte colony-stimulating factor receptor
HOXA10	Homeobox A10
hCG	Human chorionic gonadotropin
IL-10	Interleukin 10
IVF	In vitro fertilization
JAK2	Janus kinase 2
MAPK	Mitogen-activated protein kinase
NK cells	Natural killer cells
PI3K	Phosphoinositide 3-kinase
RIF	Recurrent implantation failure
SOCS3	Suppressor of cytokine signaling 3
STAT3	Signal transducer and activator of transcription 3
TGF-β	Transforming growth factor beta
Th1	T helper type 1 cells
Th17	T helper type 17 cells
Treg cells	Regulatory T cells
uNK cells	Uterine natural killer cells
